# Alpha-Synuclein Strain Variability in Body-First and Brain-First Synucleinopathies

**DOI:** 10.3389/fnagi.2022.907293

**Published:** 2022-05-26

**Authors:** Mie Kristine Just, Hjalte Gram, Vasileios Theologidis, Poul Henning Jensen, K. Peter R. Nilsson, Mikael Lindgren, Karoline Knudsen, Per Borghammer, Nathalie Van Den Berge

**Affiliations:** ^1^Institute for Clinical Medicine, Aarhus University, Aarhus, Denmark; ^2^Nuclear Medicine and PET, Aarhus University Hospital, Aarhus, Denmark; ^3^Department of Biomedicine, DANDRITE-Danish Research Institute of Translational Neuroscience, Aarhus University, Aarhus, Denmark; ^4^Division of Chemistry, Department of Physics, Chemistry and Biology, Linköping University, Linköping, Sweden; ^5^Department of Physics, Norwegian University of Science and Technology, Trondheim, Norway

**Keywords:** animal models, synucleinopathies, Lewy body disorders, seed amplification assay, oligothiophene ligands, peripheral biomarkers

## Abstract

Pathogenic alpha-synuclein (asyn) aggregates are a defining feature of neurodegenerative synucleinopathies, which include Parkinson's disease, Lewy body dementia, pure autonomic failure and multiple system atrophy. Early accurate differentiation between these synucleinopathies is challenging due to the highly heterogeneous clinical profile at early prodromal disease stages. Therefore, diagnosis is often made in late disease stages when a patient presents with a broad range of motor and non-motor symptoms easing the differentiation. Increasing data suggest the clinical heterogeneity seen in patients is explained by the presence of distinct asyn strains, which exhibit variable morphologies and pathological functions. Recently, asyn seed amplification assays (PMCA and RT-QuIC) and conformation-specific ligand assays have made promising progress in differentiating between synucleinopathies in prodromal and advanced disease stages. Importantly, the cellular environment is known to impact strain morphology. And, asyn aggregate pathology can propagate trans-synaptically along the brain-body axis, affecting multiple organs and propagating through multiple cell types. Here, we present our hypothesis that the changing cellular environments, an asyn seed may encounter during its brain-to-body or body-to-brain propagation, may influence the structure and thereby the function of the aggregate strains developing within the different cells. Additionally, we aim to review strain characteristics of the different synucleinopathies in clinical and preclinical studies. Future preclinical animal models of synucleinopathies should investigate if asyn strain morphology is altered during brain-to-body and body-to-brain spreading using these seeding amplification and conformation-specific assays. Such findings would greatly deepen our understanding of synucleinopathies and the potential link between strain and phenotypic variability, which may enable specific diagnosis of different synucleinopathies in the prodromal phase, creating a large therapeutic window with potential future applications in clinical trials and personalized therapeutics.

## Introduction

Parkinson's disease (PD), dementia with Lewy bodies (DLB), pure autonomic failure (PAF) and multiple system atrophy (MSA) are categorized as synucleinopathies, as they are all characterized by pathological accumulation of aggregated asyn protein. PD, DLB and PAF predominantly present with intraneuronal and neuritic deposits of aggregated asyn, also called Lewy pathology (Visanji et al., 2019). MSA is characterized by predominant glial cytoplasmic inclusions (GCIs), later also called Papp-Lantos bodies (Papp et al., 1989; Jellinger and Lantos, 2010). These asyn aggregates can spread through synaptically coupled networks along the brain-body axis and ultimately result in widespread neurodegeneration in the central nervous system (CNS) and in multiple peripheral organs (Beach et al., 2010; Chiang and Lin, 2019; Mendoza-Velásquez et al., 2019). The clinical representation of PD, DLB, PAF, and MSA patients is highly heterogeneous and overlapping during the early disease stages (Palermo et al., 2020; Berg et al., 2021; Folke et al., 2022). Due to the variable involvement of different neuronal systems, each synucleinopathy may include a wide range of motor, cognitive, gut and other autonomic deficits, up to 20 years prior to diagnosis, complicating early and accurate diagnosis. Diagnosis is currently made by clinical evaluation of these symptoms that can be corroborated by imaging for neurodegenerative and autonomic damage. Thus, early prodromal diagnosis remains challenging. Furthermore, patients are often misdiagnosed and can only be classified as PD, DLB, PAF, or MSA upon post-mortem investigation of the spatiotemporal distribution of pathogenic asyn in the brain (Kovacs, 2016). Thus, we are in need of early disease biomarkers for different synucleinopathies.

Recent imaging and post-mortem neuropathological evidence suggest that disease heterogeneity in PD, DLB and PAF can be explained in part by variable disease onset site: a body-first subtype where pathogenic asyn arises in the body and spreads to the brain, and a brain-first subtype where pathogenic asyn arises in the brain and spreads to the body (Horsager et al., 2020; Borghammer, 2021; Borghammer et al., 2021). On the other hand, the existence of distinct protein aggregate morphologies has also been suggested to explain disease heterogeneity in synucleinopathies (Surguchov, 2020; Holec and Woerman, 2021; Woerman, 2021). It has been shown that distinct types of synucleinopathies exhibit asyn aggregates with different morphological and biochemical traits influencing, e.g., cell tropism and toxicity of the asyn aggregate or strain (Shahnawaz et al., 2020; Van der Perren et al., 2020). Additionally, the cellular milieu in which the asyn aggregation takes place seems to significantly impact the properties of the asyn strains, ultimately altering further disease progression (Holec and Woerman, 2021).

Thus, emerging evidence suggests that disease heterogeneity either depends on disease initiation site (body or brain), or on structural characteristics of the dominant asyn strain present. However, it has not been considered that an interplay of both mechanisms could be true. Importantly, the cellular environment is known to impact pathology morphology. Thus, it is plausible that the cellular environment at the disease initiation site determines the dominant asyn strain, which then propagates through the peripheral and CNS. Here, we hypothesize, for the first time, that (1) disease initiation site and (2) pathogenic asyn conformation are both (interdependent) determinants of the clinical and histopathological profile of synucleinopathies.

Since the early disease or pre-motor phase is seen as the ideal time window for applying personalized disease-modifying therapy (Oertel and Schulz, 2016), it is crucial to establish diagnostic methods that are able to trace pathology prior to autonomic or CNS damage. Pathology in easily accessible peripheral tissues and fluids have been under investigation as a possible early biomarker for different synucleinopathies. Over the past two decades a range of asyn seed amplification assays (asyn SAA) and conformation-specific fluorescent dyes have been created to investigate the relation between the structural and seeding properties of pathology and the clinical representation of a certain synucleinopathy. The identification and characterization of disease-specific asyn aggregates in easily accessible peripheral fluids or tissues may enable early stratification as well as development of subtype-specific targets for therapeutic strategies. Especially in immunotherapy, target biology should be optimized toward strain-specific pathology (Folke et al., 2022). For this purpose, it is crucial to gain insight in the different structural characteristics of asyn strains underlying different synucleinopathies. Here, we discuss strain variability in synucleinopathies and recent developments of ultra-sensitive amplification asssays and conformation-specific dyes in neurodegenerative disease, their shortcomings and highlight the need of novel experimental models to increase translation ability of results.

## Alpha-Synuclein and Strain Variability

### Alpha-Synuclein

In 1993, a non-Aβ component of Alzheimer's disease (AD) amyloid (NAC) was discovered in homogenates of an AD brain. cDNA cloning revealed NAC was part of a NAC precursor (NACP), and sequence analysis showed NACP was a 140 amino acid protein (Uéda et al., 1993). Purification and protein sequencing of two human proteins of 140 and 134 amino acids showed the 140 amino acid protein was identical to NACP and was referred to as asyn (Jakes et al., 1994).

An A53T mutation in the asyn gene, *SNCA*, found in early-onset PD families was the first evidence linking asyn to PD (Polymeropoulos et al., 1997). Other missense mutations in addition to duplications and triplications have been identified giving rise to monogenic PD (Krüger et al., 1998; Singleton et al., 2003; Chartier-Harlin et al., 2004; Zarranz et al., 2004; Appel-Cresswell et al., 2013). Soon after asyn was linked to genetic PD, immunohistochemical studies identified asyn as a major component of Lewy bodies (LBs) and Lewy neurites in PD and DLB (Spillantini et al., 1997), and of GCIs in MSA (Tu et al., 1998).

Physiological asyn is present in abundance in presynaptic terminals of neurons and is thought to be involved in synaptic transmission, dopamine metabolism and lipid vesicle trafficking (Murphy et al., 2000). Under normal physiological conditions, asyn exists in equilibrium between membrane-bound and soluble native states. In pathological conditions, asyn can misfold from its native soluble conformation into different β-sheet enriched structures (oligomers or protofibrils), ultimately forming elongated insoluble fibrils that accumulate into Lewy-like aggregates (Lashuel et al., 2013). What allows asyn misfolding to take place is currently unknown, an interplay of several mechanisms may take place, such as increased amounts of endogenous asyn, the presence of asyn mutations, cellular stress, mitochondrial dysfunction, asyn aggregate inducing proteins and metabolites (Lindersson et al., 2005; Lee et al., 2011; Mehra et al., 2019).

This aggregation process can be replicated *in vitro*, using recombinant asyn, reproducing different fibrillary asyn structures resembling asyn aggregates isolated from human from samples. Candelise et al. have elaborately reviewed the micro-environmental factors that influence asyn aggregation. Briefly, chemical factors such as low pH has been shown to promote aggregation alongside with increased temperature, shaking and presence of metal ions. Furthermore, physiological variations in the cellular organelles such as pH, and biological factors such as fatty acids may influence aggregation. For example, some short-saturated hydrocarbon chains are able to induce asyn aggregation opposed to polyunsaturated fatty acids that quench aggregation (Candelise et al., 2020).

### Seeding

A misfolded asyn protein can recruit endogenous asyn and induce pathogenic conformational changes in the endogenous protein, resulting in conformations of asyn with high content of β-sheets, gradually building up to insoluble pathogenic conformers called fibrils. This mechanism is defined as conformational templating, also called “seeding”, and was originally discovered in prion diseases. In 1982, when studying the scrapie protein properties, Prusiner proposed the term prion to describe a small proteinaceous infectious particle (Prusiner, 1982). The prion protein (PrP^c^) is the pathological hallmark of transmissible spongiform encephalopathies (TSEs). TSEs cover a range of neurodegenerative diseases in mammalians including among others: scrapie (sheep), chronic wasting disease (CWD) (deer), bovine spongiform encephalopathy (cattle), Creutzfeld-Jakob disease (CJD), Gerstmann-Sträussler-Scheinker disease, fatal familial insomnia and kuru (human). Characteristic of TSEs is the infectious properties of PrP that either sporadically or upon interaction with a pathological PrP^Sc^ (scrapie prion protein) undergo conformational changes from a native state of alpha-helical structure into pathological β-sheet-enriched amyloid aggregates. Importantly, conformational templating is not limited to one cell. The spreading of prions is thought to occur through cell-to-cell propagation *via* exocytosis, endocytosis, interstitial diffusion and tunneling nanotubes (Jaunmuktane and Brandner, 2020).

It is hypothesized that, similar to prions, amyloid and misfolded asyn possess seeding properties, can propagate *via* cell-to-cell transmission using similar mechanisms, and are neurotoxic (Guo and Lee, 2014). The one thing differing the non-prion proteins of neurodegenerative diseases from PrP^Sc^ so far lies in the infectious properties of PrP^Sc^. Extensive research has demonstrated seeding and propagation ability of patient-extracted asyn and artificial fibrillar asyn upon inoculation in various *in vivo* and cell models (Bernis et al., 2015; Prusiner et al., 2015; Woerman et al., 2015; Kim et al., 2019; Van Den Berge et al., 2019, 2021; Van Den Berge and Ulusoy, 2022), including demonstration of serial propagation (Watts et al., 2014; Woerman et al., 2019), but there is currently no evidence of human-to-human transmission of asyn aggregate dependent disease (Jaunmuktane and Brandner, 2020).

### The Strain Hypothesis

The strain hypothesis in synucleinopathies stems from the prion hypothesis, originally defined in the context of prion disorders. The prion hypothesis or protein-only hypothesis states that misfolded proteins with the capability to recruit physiologically homologous proteins in their native state and induce conformational change leading to prion pathology with a distinct structure are the underlying cause of distinct phenotypes in prion disorders. These distinct structures are called “strains”. The earliest evidence of strains came from a study in 1961 by Pattison and Millson, who proposed a relationship between the inoculum and clinical phenotype of scrapie in goats where some strains of the scrapie agent led to the nervous syndrome and others to the scratching syndrome (Pattison and Millson, 1961). In humans, CJD was the entry of strains in regards to terminology due to the identification of prion proteins with various glycosylations in distinctive clinical phenotypes showing differential anatomical distribution patterns of pathology. A detailed review on the concept of prion strains has been provided by Collinge and Clarke (2007).

Extensive research demonstrates the extension of the prion hypothesis to other neurodegenerative disease, including synucleinopathies, but also Alzheimer's disease (AD) and tauopathies, reviewed by Walker and Jucker (2015). Here, we briefly discuss the main evidence of prion properties and existence of strains in synucleinopathies. In 1998, Grazia Spillantini et al. demonstrated asyn filaments from MSA patients that were either straight or twisted. These filaments were not found in control brains (Grazia Spillantini et al., 1998). Similarly, filaments of tau in various tauopathies also showed straight or twisted characteristics, applicable for tau filaments of the isoforms 3R, 4R or a combination of the two (Crowther and Goedert, 2000). In 2013, Bousset et al. generated two polymorphs *in vitro* that differed structurally and functionally. The polymorphs were denominated fibrils and ribbons, respectively, and the researchers showed that fibrils were more toxic than ribbons (Bousset et al., 2013). In continuation hereof, researchers have investigated the properties of ribbons, fibrils and oligomers. In animal models it was reported that fibrils were more toxic than oligomers, which was an unexpected finding in light of oligomers being the commonly thought conformer to have the most toxic properties (Peelaerts et al., 2015). In post-mortem brains, the filamentous aggregates could be amplified and resembled the *in vitro* reference strains ribbons and fibrils, both separately and mixed in different anatomical regions within individual brains as demonstrated by transmission electron miscroscopy (TEM) and proteolytic profiling (Fenyi et al., 2021).

In 2015, Prusiner et al. did a remarkable study showing that asyn extracted from MSA patient brains was able to seed pathology in cells and animals, and hereby provided evidence that asyn is a prion (Prusiner et al., 2015). In their study neither brain extracts from controls or PD patients were able to induce pathology in the *in vivo* models used, indicating that asyn in MSA is a distinctive strain from asyn in PD. A similar study was conducted by Bernis et al. (2015) revealing propagation of asyn extracted from MSA and probable incidental Lewy Body Disease (LBD) brains upon inoculation into the striatum of transgenic mice.

An elaborate study on the existence of asyn strains in synucleinopathies was conducted by Van der Perren et al. They extracted asyn from the brains of MSA, PD, DLB patients and controls in order to investigate the characteristics of asyn in regard to structure, seeding propensity, as well as propagation and neurotoxicity *in vivo* using cell and rodent animal models (Peng et al., 2018; Van der Perren et al., 2020). Interestingly, the authors reported that MSA-derived asyn is characterized by a more aggressive seeding in *in vivo* models in line with the clinical pathophysiology of MSA compared to the other synucleinopathies (Van der Perren et al., 2020). In MSA, three strains of distinct Morphologies, type 1–3, have recently been characterized *via* cryo-EM (Schweighauser et al., 2020; Lövestam et al., 2021).

As previously mentioned, the cellular environment can affect the conformations of asyn. A study on asyn strains in LBD and MSA reported that the cellular milieu of oligodendrocytes and neurons, respectively, generates different asyn strains (Peng et al., 2018). However, cell type specific preferences of asyn in GCIs and LBs were not demonstrated. In contrast, carboxy truncated asyn in MSA and DLB did show cell type specific preferences (Hass et al., 2021).

The ability of specific asyn strains to produce distinct disease phenotypes in animal models is under investigation. Two recent studies test this hypothesis by generating distinct asyn fibrils under various conditions and by inoculating these strains at different sites (intracerebral or intramuscular). Both studies reported phenotypic variations associated with strain morphology. Phenotypic variations include variations in incubation time, symptoms, degree of pathology as well as affected regions and cell types representing various pathological profiles (Lau et al., 2020; Liu et al., 2021). These findings support the notion of specific asyn strains causing distinct disease phenotypes.

Finally, examination of post-mortem brains has revealed co-occurrence of asyn, tau and amyloid-beta (Aβ) in tauopathies and synucleinopathies which have led to the concept of cross seeding. It is thought that interaction between the aggregation prone proteins may synergistically promote aggregation of these. *In vivo* and *in vitro* studies have demonstrated that cross seeding of asyn, tau and Aβ is possible (Götz et al., 2001; Giasson et al., 2003; Guo et al., 2013; Williams et al., 2020). However, it is not without difficulties to study cross seeding as factors like tissue stress may affect aggregation. Consequently, mechanisms in cross seeding remain to be elucidated.

## Strain Variability Along the Brain-Body Axis—a New Hypothesis

### The SOC Model

Emerging data from post-mortem and imaging studies suggest that heterogeneity in PD could be explained by variable disease onset sites (brain vs. body) and by aspects of the brain connectome. This has been termed the synuclein origin and connectome (SOC) model (Borghammer, 2021). The model proposes two overall subtypes of PD: (1) a brain-first type, where pathology initially appears in a single hemisphere in the brain (usually the amygdala or olfactory bulb). Initial propagation of synuclein pathology occurs primarily *via* ipsilateral connections, which strongly outnumber contralateral (commissural) projections, leading to asymmetric dopamine loss, asymmetric parkinsonism, and secondary spreading to the peripheral autonomic nervous system (ANS); (2) a body-first type, where the pathology originates in the peripheral ANS (usually the gut), causing early cardiac and enteric dysfunction, and then spreads to the brain bilaterally *via* left-right overlapping vagal and sympathetic projections. This results in more symmetric initial involvement of the CNS leading to more symmetric dopamine loss and more symmetric parkinsonism (Horsager et al., 2020, 2022; Borghammer et al., 2021).

These hypotheses are based on imaging data obtained in newly diagnosed PD patients with and without REM-sleep behavior disorder (RBD). RBD is characterized by dream enactment and REM-sleep without atonia, due to damaged pontine structures, including the locus coeruleus, subcoeruleus, and pedunculopontine tegmental nucleus. Pre-motor or isolated RBD (iRBD) is therefore considered a marker of (prodromal) body-first PD as ascending pathology affects pontine structures prior to involvement of the SN. Importantly, 80% of iRBD cases pheno-convert to PD or DLB within a decade (Meles et al., 2021). By contrast, in later stage brain-first PD, RBD develops after motor symptoms as ultimately pathology will descend from the SN to lower Braak stage structures in these patients, causing RBD (Borghammer and Van Den Berge, 2019; Andersen et al., 2020; Horsager et al., 2020). In iRBD patients and newly diagnosed PD patients with RBD, Horsager et al. have shown cardiac and enteric denervation, measured with ^123^I-MIBG scintigraphy and ^11^C-donepezil PET, respectively, prior to nigrostriatal dopamine deficit, measured with ^18^F-FDOPA positron emission tomography (PET), in support of the body-first trajectory. In contrast, they observed asymmetric dopamine deficit prior to cardiac and enteric denervation in newly diagnosed PD patients without RBD, representing brain-first PD (Horsager et al., 2020, 2022; Knudsen et al., 2021). Ultimately, all patients converge on a similar advanced disease stage phenotype, where heart, gut, and brain are all affected. In addition, a recent review of several neuropathological studies indicate the existence of the same subtypes across several Lewy body cohorts, as patients could be categorized as (1) a brainstem-predominant subtype with more severe pathology in the intermediolateral nucleus of the spinal cord (IML) and sympathetic ganglia, possibly explaining early autonomic dysfunction in this subtype, or (2) an amygdala/limbic-predominant subtype with minimal or no pathology in autonomic structures at early disease stages. These patterns seem to be representative of a body-first and brain-first PD trajectory (Borghammer et al., 2021).

### The SOC Model Applied to the Full Lewy Body Spectrum

The SOC model applies to the full spectrum of Lewy body disorders (LBDs), thus also includes PAF and DLB (Borghammer, 2021). PAF is a sporadic neurodegenerative disorder characterized by failure of the autonomic system and the presence of peripheral pathology in the sympathetic ganglia and in axons of autonomic neurons, incl. the heart, bladder, skin, and colon, post-mortem (Hague et al., 1997) and *in vivo* (Donadio et al., 2016). Importantly, asyn inclusions in skin biopsies from PAF patients appear to be indistinguishable from those seen in PD (Donadio et al., 2016). Moreover, the majority of PAF patients show severe loss of sympathetic cardiac innervation on ^123^I-MIBG scintigraphy or ^18^F-dopamine PET (Goldstein et al., 2011; Donadio et al., 2016), which is also typical of many PD and DLB patients. Furthermore, LBs are seen in brainstem nuclei, including the locus coeruleus and SN in most patients at autopsy (Hague et al., 1997), demonstrating that PAF can be considered a prodromal body-first LBD. Recent follow-up studies show that a large fraction of PAF patients pheno-convert to PD, DLB or MSA, and those who do, nearly always have RBD, a manifestation of body-first LBD (Kaufmann et al., 2017). Note, here we include PAF as a LBD, as we are referring to asyn-inclusion-positive PAF patients. The fraction of asyn-synuclein inclusion-positive PAF patients among all PAF patients is currently unknown, but several studies suggest that it could be the majority of PAF patients (Donadio et al., 2016), with more rare cases being caused by other pathologies such as autoimmune autonomic ganglionopathy (Coon and Low, 2017).

The clinical representation of DLB is very similar to PD, and differential diagnosis is based on the occurrence of early cognitive dysfunction with an onset <1 year after appearance of motor dysfunction (Armstrong and Emre, 2020), which is a very short time, considering that non-motor symptoms occur up to 20 years prior to motor symptoms in some Lewy body patients (Borghammer, 2018). Similar to PD the majority of DLB patients show dopaminergic degeneration in the substantia nigra (Tsuboi et al., 2007). Importantly and perhaps surprisingly, most DLB patients exhibit the full spectrum of symptoms and findings associated with the body-first subtype. Compared to PD, newly diagnosed DLB patients have more frequent hyposmia and iRBD, more frequent pathological MIBG heart scans, and more symmetric nigrostriatal degeneration on brain imaging. It is believed that DLB patients, compared to strict body-first PD, show more widespread pathological changes throughout the neocortex and limbic system (Jellinger and Korczyn, 2018). Importantly, 60–90% of DLB patients appear to have co-existent AD pathology, which may influence the pattern of asyn pathology and contribute to even faster cognitive decline. Therefore, we speculate that DLB is often caused by the presence of two separate neurodegenerative disorders that progress in parallel but which may also show deleterious synergistic effects, namely strict body-first LBD and co-existent AD. While PD patients are diagnosed around 65 years of age, on average, DLB patients are diagnosed around 75 years of age. Importantly, 25–30% of the healthy elderly above 75 years old exhibit AD pathology in their brain (Jansen et al., 2015). We speculate that body-first LBD in an Aβ-null brain leads to strict body-first PD (70–75% of cases), which nevertheless shows fast progression toward dementia (PDD or DLB), but that body-first LBD in an already Aβ-positive brain may cause even faster cognitive decline often resulting in the DLB-phenotype (25–30% of cases). Furthermore, it is important to note that asyn, Aβ and tau protein aggregates are both able to cross-seed, i.e., template and aggravate the other pathology (Lim, 2019; Williams et al., 2020). Thus, both body-first PD and DLB are considered body-first LBDs, and differences in phenotype and neuropathological findings may be attributed to co-existent AD pathology in DLB cases.

**Figure 1:**
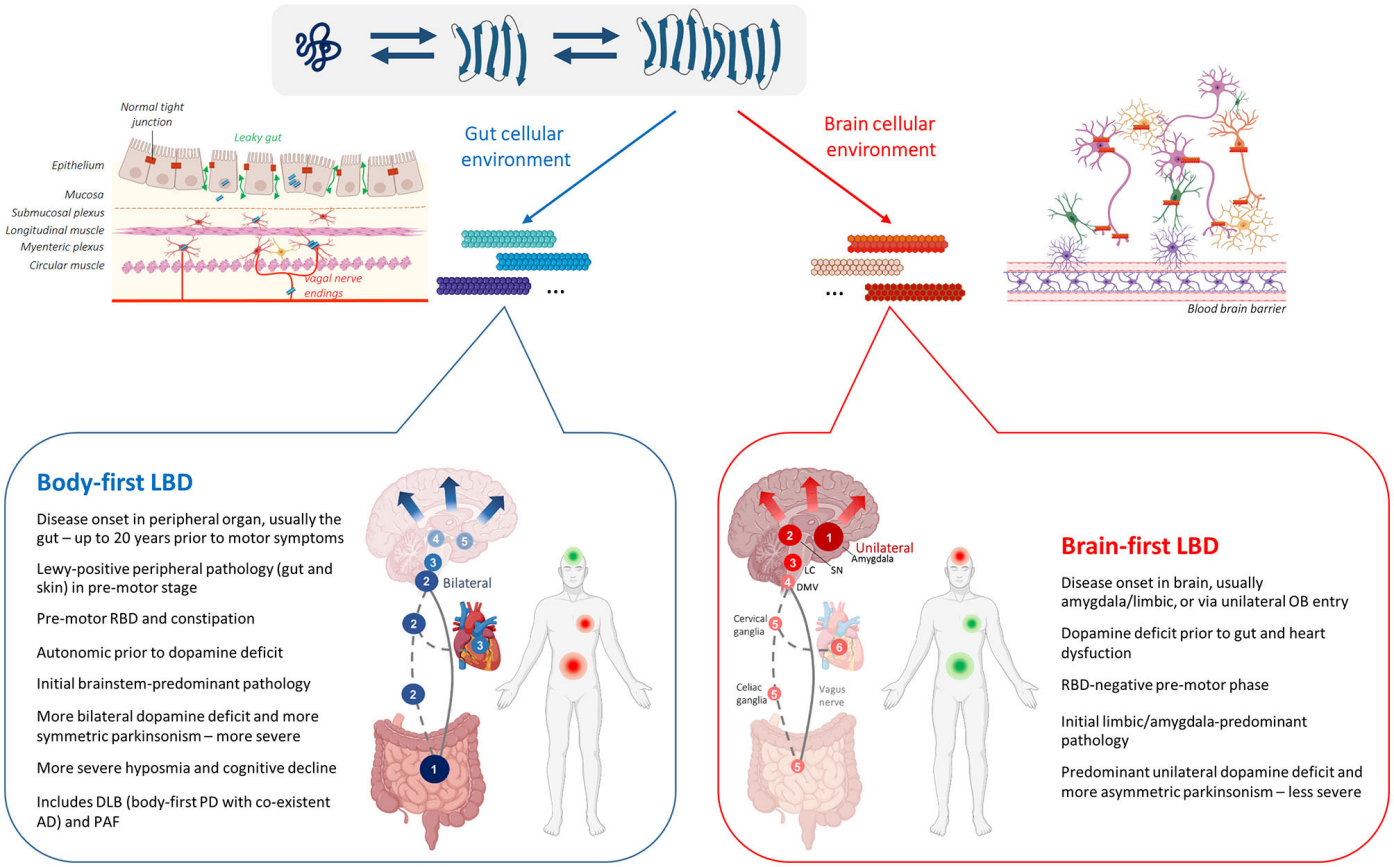
Asyn strain variability in the LBD asyn origin and connectome (SOC) model. **Upper panel**: Schematic representation of the strain hypothesis in LBD as described by the SOC model. Physiological unfolded monomeric protein can misfold into pathogenic β-sheet-rich subunit conformations that are capable of seeding other pathogenic subunit conformers or monomers, ultimately elongating into mature fibrillary asyn. Monomeric asyn can adopt various β-sheet-rich conformations depending on the cellular milieu where the misfolding occurs. Therefore, distinct fibrillary asyn conformers or 'strains', composed of different subunits, can arise depending on the cellular environment, determined by the neuron-type and its location (body or brain). Each distinct asyn strain may affect different parts of the brain and autonomic connectome in varying degrees, contributing to the heterogenous clinical representation of body-first and brain-first LBDs. **Lower panel**: Schematic representation of two LBD-subtypes as predicted by the SOC model. Numbers indicate progression of pathology from initiation (1) to more advanced disease stages (4–6). In body-first LBD, asyn arises in the ANS (usually the gut) from where it spreads bilaterally via overlapping vagal (full line) and sympathetic (dashed line) connections to the brain and to other peripheral organs causing autonomic dysfunction prior to dopaminergic deficit. Bilateral invasion of pathology in the DMV causes subsequent bilateral involvement of the LC causing RBD, and bilateral nigrostriatal neurodegeneration with symmetric parkinsonism as disease further progresses. In brain-first LBD, asyn arises unilaterally in the amygdala or olfactory bulb, after which it spreads to the unilateral SN causing unilateral neurodegeneration and asymmetric parkinsonism. Upon further progression to the LC, RBD may occur post motor deficit. Inevitably, pathology spreads to peripheral organs, causing autonomic dysfunction at advanced disease stages. Due to the low level of homotypic connections in the brain, initial predominant unilateral pathology and associated neurodegeneration is initially confined to a single hemisphere for some time in brain-first synucleinopathy. In contrast, bilateral invasion of pathology in the brain of body-first cases causes a more severe disease progression, including more severe hyposmia and cognitive decline. Body-first and brain-first LBD mainly predict disease progression in PD patients with and without pre-motor RBD. Furthermore, most DLB and all PAF patients can be categorized as having body-first LBD. Although cellular vulnerability and the presence of concomitant AD pathology may influence the spatial distribution of pathology and clinical profile in these LBDs compared to typical body-first PD. Abbreviations: SN, substantia nigra; LC, locus coeruleus; DMV, dorsal motor nucleus of the vagus; RBD, REM-sleep behavior disorder; DLB, dementia with Lewy bodies (LBs); PAF, pure autonomic failure (Created with Biorender).

Each LBD progresses at different velocities and with different intensities, but all eventually reach a similar late disease stage where the entire body is affected (Borghammer and Van Den Berge, 2019; Borghammer, 2021). Figure 1 summarizes the body-first and brain-first LBD as described in this review.

### The Strain Hypothesis in Synucleinopathies

MSA patients present mainly with GCIs (which consist mainly of asyn) and only minor low-density spread of neuronal inclusions, and is therefore considered a synucleinopathy, but not a LBD (Cykowski et al., 2015). Multiple studies have shown that MSA-derived asyn and LBD-derived asyn are conformationally and biologically distinct, hereby linking a certain asyn strain conformation to the clinical representation of a certain synucleinopathy (Peng et al., 2018). It is hypothesized that the cellular environment in oligodendrocytes or neurons favors one conformation over another, consequently creating an MSA-specific and LBD-specific strain, respectively (Woerman, 2021).

In brain-first LBD, pathogenic asyn could theoretically arise stochastically in several different types of neurons in almost any brain region, possibly resulting in a region or neuron-specific strain. A brain region contains several other cell-types including microglia, astrocytes, and oligodendrocytes, which may influence asyn aggregation (George et al., 2019). The neurons in the brain are protected by the blood-brain-barrier (BBB), composed of endothelial cells, to shield the brain from toxic substances circulating in the blood. In the case of body-first LBD, pathogenic asyn may arise in the ANS, usually in the enteric neurons of the gut or in the parasympathetic or sympathetic terminals and axons innervating those enteric neurons. The gut wall provides a very different cellular environment compared to the brain. It is not protected by a blood-brain-barrier and needs a complex cellular organization to prevent foreign pathogens from entering the host. Therefore, the gut wall possesses a complex mix of different cell-types, including neurons, glia, endocrine, immune and epithelial cells, that are meticulously organized in concentric layers to regulate gut motility, blood flow, and secretion. If a distinct asyn strain conformation is preferentially selected by a certain neuron-type in a certain brain region, it is conceivable that this also occurs in the gut with an even more complex cellular organization that additionally may undergo alterations caused by the microbiome. Thus, here we hypothesize that the clinical representation in body and brain-first LBDs, in addition, could be explained through preferential selection of a certain conformer by the cellular milieu at the disease onset site. Figure 1 represents a graphical representation of this hypothesis in the LBD-SOC model framework.

Similar to LBDs, both the CNS and ANS are affected in MSA patients. In case MSA presents with solely autonomic dysfunction, patients can be initially diagnosed with PAF, whereas MSA patients presenting with parkinsonism can be misdiagnosed as PD (Palma et al., 2018). In fact, definite diagnosis of MSA is reliant on post-mortem detection of widespread GCIs in oligodendrocytes (Gilman et al., 2008). MSA seems to share some characteristics with body-first LBD as it presents with bilateral neurodegeneration, symmetric parkinsonism, a severely damaged brainstem, variable autonomic dysfunction and a very rapid disease progression (5–10 years) (Batla et al., 2013). However, until now there is no evidence indicating MSA-pathology may start outside the brain, as MSA cases do not show obvious pathology in the peripheral nervous system, and present with normal MIBG scintigraphy of the heart (Braune et al., 1999). The MSA pathology is mainly located to preganglionic autonomic neurons, such as in the IML (Jellinger, 2020). In contrast, in PD it is known that both the pre- and post-ganglionic neurons are severely involved and all LBD cases eventually develop pathological MIBG (Sulzer and Surmeier, 2013; Horsager et al., 2020).

**Figure 2:**
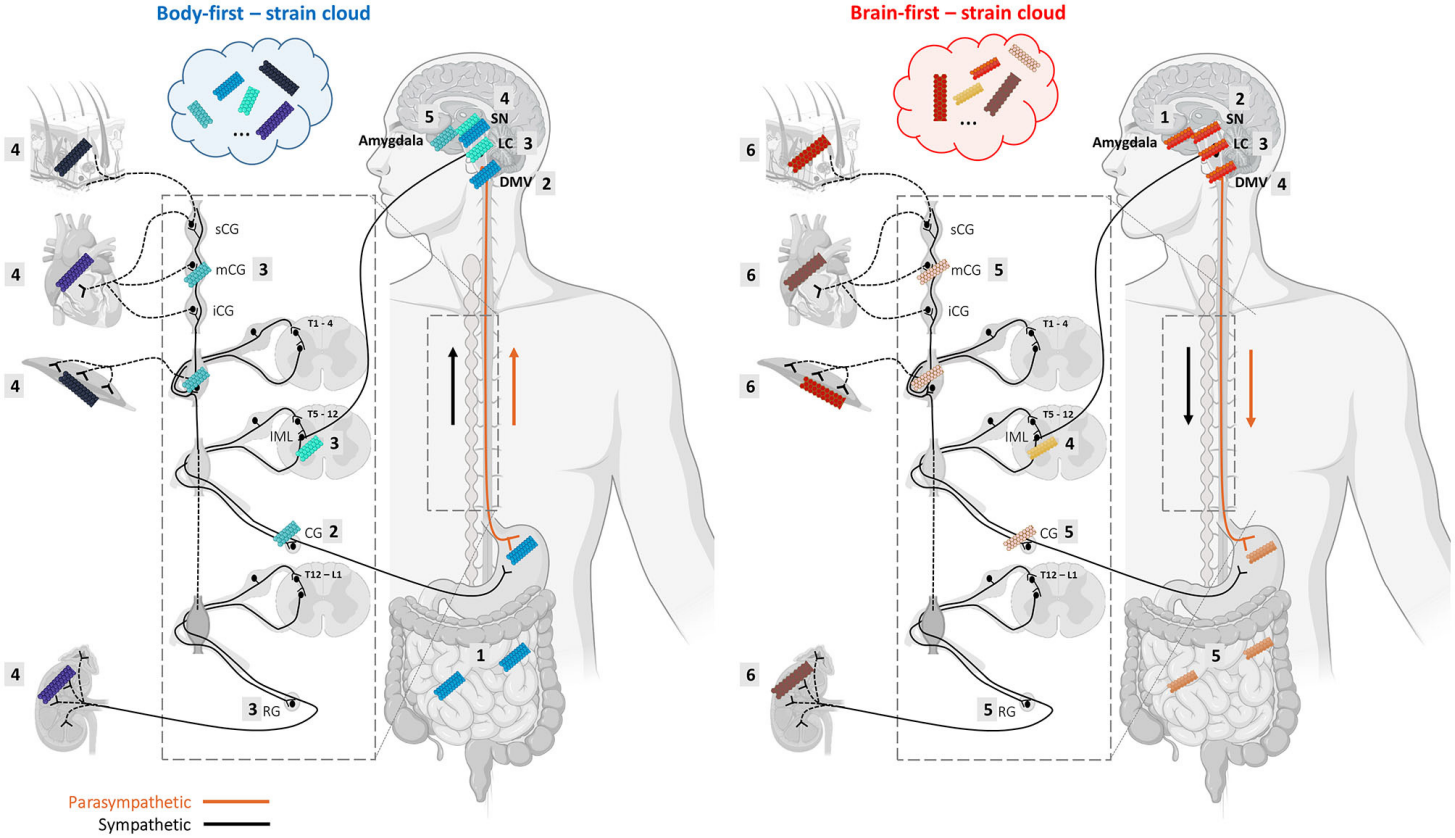
Alpha-synuclein strains may change morphology during trans-synaptic spread along the body-brain axis, resulting in a mix of different conformers in body-first and brain-first LBDs. Monomeric asyn can adopt different β-sheet-rich conformations depending on which conformation is favored by the cellular milieu, which is defined by a certain cell-type and organ where the seeding takes place. The changing cellular environment during trans-synaptic spread along the body-brain axis may give rise to the formation of different asyn conformers throughout the connectome, and even within a single organ. Thus, despite strain characteristics appear to be linked to a certain LBD-subtype, heterogeneity within a subtype may be caused by different dominating strains in their “strain cloud”. The numbered squares indicate the stages of pathology progression throughout the ANS in body-first **(left)** and brain-first **(right)** LBD. We speculate that body-first LBD may give rise to a more variable strain cloud, since pathology can initiate in any peripheral organ and enter the brain *via* parasympathetic as well as sympathetic connections. This might explain why body-first LBDs exhibit distinct disease profiles within (i.e., typical body-first PD, DLB, and PAF). Importantly, propagation of pathology may occur bidirectionally, possible creating even more strain variations (Created with Biorender).

The vast majority of preclinical studies of synucleinopathies use a brain-initiated disease model and focus on CNS pathology and neurodegeneration. In the past decade, an increasing number of studies aim to model body-first LBD by initiating pathology in the gut (Pan-Montojo et al., 2010; Holmqvist et al., 2014; Kim et al., 2019; Van Den Berge et al., 2019, 2021) or autonomic ganglia (Wang et al., 2020a), and include other important body-first features such as peripheral pathology and autonomic dysfunction (Van Den Berge and Ulusoy, 2022). These studies have shown that bidirectional body-to-brain and brain-to-body spread may occur along vagal as well as sympathetic connections, affecting several structures with different cell-types along the way, including the IML, autonomic ganglia, heart, gut, skin, muscle, kidney, liver, spleen, and adrenal glands. All these structures are characterized by a different cellular organization and milieu. It is thus plausible that trans-synaptic seeding of pathology across the autonomic connectome may favor a specific conformation over another at each “station”, ultimately resulting in accumulation of multiple strains within one subtype (see Figure 2). Importantly, different CWD prion strains have been characterized (with different amplification properties) in the muscle tissues of tongue, neck, hindlimb, forelimb, backstrap, and tenderloin of white-tailed deer (Li et al., 2021). Furthermore, Rasmussen et al. (2017) have shown that individual patients with AD can display several distinct types of conformational variants of Aβ, and that the mix of amyloid-conformers and its distribution within the “amyloid strain cloud” may vary among familial and non-familial AD subtypes. In LBDs, the “asyn strain cloud” may be even more variable due to differential preference of a certain conformer at the onset site (enteric neuron or autonomic ganglion neuron in body-first LBD vs. several different neuron types in brain-first LBD with amygdala-neurons being the most common).

Future studies should include the investigation of subtype-specific strain characteristics in different animal and patient studies to investigate the relation of strain development along the brain-body axis with disease onset site and phenotype. The long prodromal phase of LBDs provides a possible window of early intervention, though this necessitates methods for early diagnosis, before pathology causes extensive damage and symptoms. The gut and skin, as well as blood, stool, and cerebrospinal fluid (CSF) samples are easily accessible to detect and quantify (subtype-specific) asyn. Unfortunately, the currently available asyn antibodies distinguish morphology poorly as they detect all forms of asyn (phosphorylated, fibrillary and oligomeric) (Kumar et al., 2020). Using ultra-sensitive asyn SAA and luminescent conjugated oligothiophenes (LCO) assays on these biopsies, different subtype-specific strains could be detected and contribute to an *a priori* screening and stratification of patients with toxic prion-like asyn phenotype.

## ASYN Amplification Assay to Identify Different Strains

Asyn aggregate seeds in easily accessible tissue and body fluids have in recent years been investigated as a possible biomarker for synucleinopathy. The prodromal phase of LBDs may initiate up to decades prior to clinical diagnosis, and circulating asyn oligomers can be detected in the CSF of LBD patients. This led the way for an assay that could identify asyn seeds in the CSF of individuals (Fairfoul et al., 2016; Shahnawaz et al., 2017; Groveman et al., 2018). To detect circulating proteopathic seeds, an asyn SAA was developed also referred to as asyn protein misfolding cyclic amplification (PMCA) and asyn real-time quaking-induced conversion (RT-QuIC). The assay, originally developed for detection of PrP^Sc^, can amplify and thereby detect trace amounts of asyn seeds in biological samples of patients using the prion-like self-propagation mechanism of asyn aggregate seeds (Fairfoul et al., 2016; Shahnawaz et al., 2017).

### Alpha-Synuclein Seed Amplification Assay

**Figure 3:**
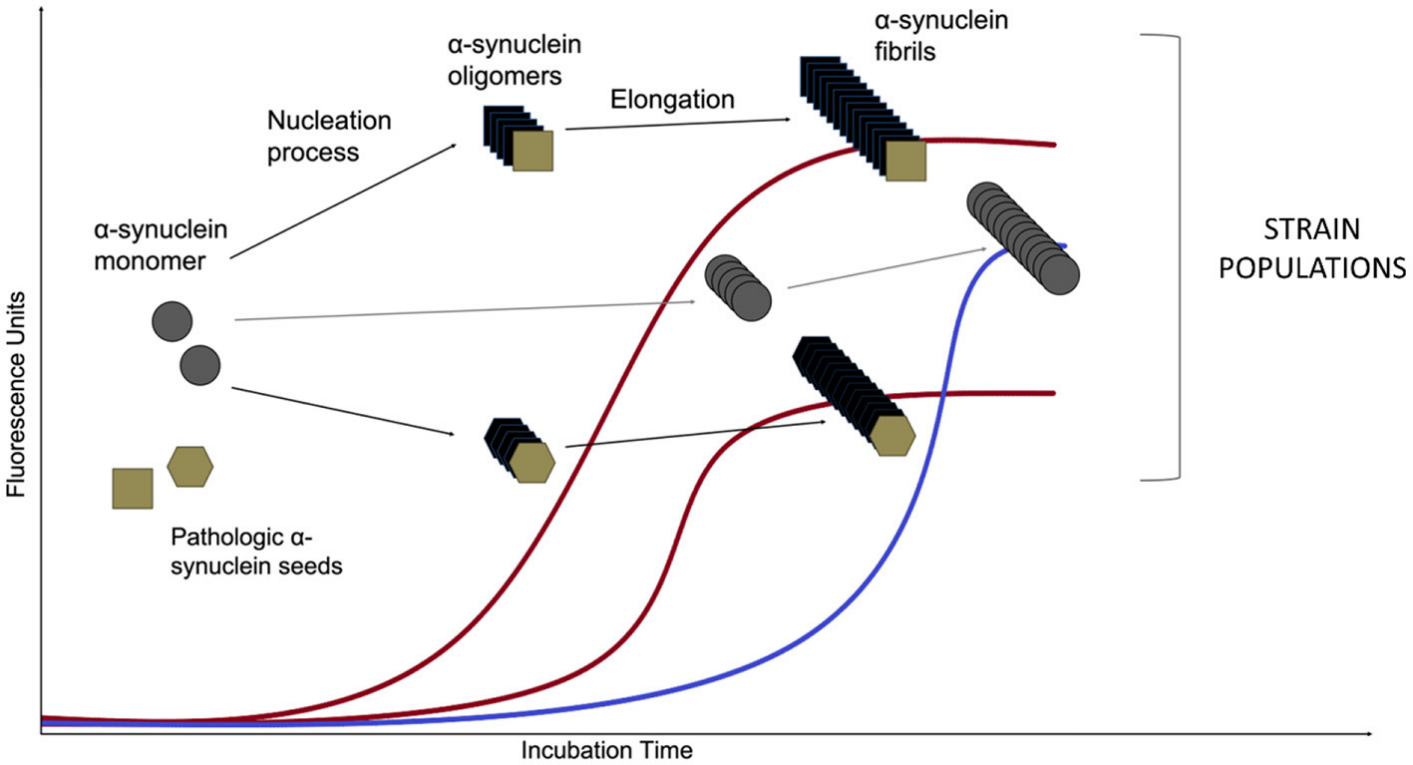
Asyn aggregation in a SAA reaction. Monomeric recombinant asyn substrate is added to the wells of a multi-well plate, along with the amyloid-selective fluorescent dye Thioflavin T and the biospecimen to be tested for the presence of asyn seeds. After an initial lag phase, the monomeric asyn can form *de novo* aggregates (so-called oligomers) that further elongate to form amyloid type β-sheet-rich fibrils during the exponential phase (the *de novo* pathway is marked in blue). The presence of structurally different asyn seeds, e.g., from PD and MSA patients (Shahnawaz et al., 2020) lead to structurally different asyn conformers, called strains, that can be detected in real time due to the ability of dyes (such as Thioflavin T and K114) that emit fluorescence when bound to amyloid structures. Distinct strains present different conformations and subsequently, altered affinity for binding of the fluorescent molecules. That leads to variations of maximum fluorescent values. The *de novo* aggregation kinetics are usually characterized by an extended lag phase when compared to the seeded aggregation and this difference forms the basis for diagnosing a biospecimen positive or negative for the presence of asyn seeds.

An asyn SAA reaction consists of an excess of the normal monomeric protein in a buffer that also contains the amyloid-specific fluorescent dye Thioflavin T (ThT). The mixture then is added into wells of multi-well plates along with the sample of interest for the putative seeding activity (illustrated in Figure 3). The incubation period is divided in cycles of shaking and rest: shaking creates mechanical force in order to break fibrillar aggregates into multiple smaller seeds and favors the interaction between the seeds and the monomer substrate, while resting allows seed elongation with the monomeric asyn. This leads to exponentially growing amounts of aggregating seeds as long as there is enough normal protein during the next cycle. Frequent measurements of ThT fluorescence provides real-time quantification and reaction kinetics of the amyloid aggregate formation (Wilham et al., 2010; Concha-Marambio et al., 2019; Saijo et al., 2019). The kinetics of amyloid aggregate formation is characterized by an initial lag phase followed by an exponential growth phase that reaches a plateau when the pool of soluble protein has been depleted. While the asyn SAA methodology is relatively simple it is not trivial. As asyn has an inherent propensity for forming *de novo* aggregates, the aim is to create a setup that favors seeded aggregation while simultaneously disfavors *de novo* aggregation.

The exact nature of these asyn seeds is still unknown, though immunodepleting asyn in patient CSF show reduced seeding potential (Shahnawaz et al., 2017), which corroborates the hypothesis that the templating-active seeds indeed contain asyn. How asyn seeds are organized is yet unknown but they must have a size that allows them to present different conformations that become imprinted into the asyn fibrils they seed (Shahnawaz et al., 2020).

The first asyn SAA, initially named Protein misfolding cyclic amplification (PMCA), was described by the Soto lab in 2001 for PrP^Sc^. The assay required repeated cycles of sonication and incubation of a sample seeded into a substrate with excess amounts of PrP^c^ amplification (Saborio et al., 2001). The prion amplification could keep going until the substrate ran out. Serial PMCA (sPMCA) overcame the limitation of definite substrate with the addition of a re-dilution step of the reaction product at the end of each cycle in a new substrate, followed by a new cycle (Castilla et al., 2005). The production of the PrP^c^ substrate used for these techniques was expensive and the amount was sometimes insufficient, as it was isolated from cells. In 2007, Caughey et al., used recombinant PrP (rPrP), produced in bacteria, as a PMCA substrate for the first time, and named this method rPrP-PMCA (Atarashi et al., 2007). Detection of aggregates using these assays was time consuming, as it was performed by proteinase K digestion and subsequent Western immunoblotting. In the same year, Prusiner et al. proposed an amyloid seeding assay (named ASA) that could detect PrP^Sc^ at femtogram level in brain samples within 1 day. The detection sensitivity was increased with the addition of ThT, a dye that displays enhanced fluorescence when bound to β-sheet-rich structures of amyloid aggregates (Colby et al., 2007). In 2008, Atarashi et al. replaced the sonication step of the PMCA assay with shaking at higher temperature. This technique was named quaking induced conversion (QuIC) and was effective for the detection of PrP^Sc^ in animals' CSF (Atarashi et al., 2008). Finally, the advantages of ASA and QuIC were combined in a rapid, sensitive and quantifiable method RT-QuIC for the detection of prion seeds in CSF (Wilham et al., 2010; Atarashi et al., 2011).

Herva et al. (2014) have shown that asyn fibrils can be used as seeds in PMCA. In addition, Jung et al. (2017) amplified asyn seeds from tissue samples with asyn-PMCA. The first CSF-based asyn RT-QuIC assay has been reported by Green et al. The assay that lasted around 5 days to be completed showed 95% sensitivity for PD and 92% for DLB, with 100% specificity in regard to AD and other neurodegenerative diseases (Fairfoul et al., 2016). Soto et al. developed the CSF-based PMCA to distinguish PD patients from individuals affected by other neurologic diseases that served as controls, with 89% sensitivity and 97% specificity for PD. This assay takes around 13 days to perform (Shahnawaz et al., 2017). In 2018, Sano et al. described an asyn-RT-QuIC assay for DLB brain tissue in <4 days (Sano et al., 2018). In the same year, Caughey et al. optimized asyn-RT-QuIC reaction conditions for CSF specimens to be completed within 1–2 days with 93% sensitivity and 100% specificity (Groveman et al., 2018).

### Recent Patient Studies and Prognostic Potential

The asyn SAA assay has been successfully employed to detect and amplify asyn seeds from a range of different biological samples. Though CSF has been most studied (Fairfoul et al., 2016; Shahnawaz et al., 2017, 2020; Groveman et al., 2018; Kang et al., 2019; Poggiolini et al., 2022), extracts of brain (Groveman et al., 2018; Bargar et al., 2021), skin (Wang et al., 2020b; Kuzkina et al., 2021), olfactory mucosa (De Luca et al., 2019), submandibular glands (Manne et al., 2020) and gut (Fenyi et al., 2019) have all been utilized to differentiate PD patients from other synucleinopathies and controls. Although there is currently no universal protocol, reports have shown high sensitivity and specificity of the assay with differing experimental conditions. A recent study showed similar results (sensitivity ranging from 86 to 96% and specificity from 93 to 100%) across three different labs utilizing the same CSF samples from PD patients and healthy controls in their respective established asyn SAA protocols (Russo et al., 2021).

The ability to detect asyn aggregates in the prodromal phase of synucleinopathies is an especially enticing aspect of the assay. In body-first PD, idiopathic RBD and PAF indicate a risk for developing PD (Iranzo et al., 2013; Coon et al., 2019). In a study of CSF from 18 iRBD and 28 PAF patients, samples were tested with asyn SAA and the results showed that the seeding activity of asyn in these samples can be detected with a sensitivity of 100 and 92.9%, respectively (Rossi et al., 2020). In another study of patients with iRBD, researchers were able to detect asyn seeds in the CSF with 90% specificity and 90% sensitivity vs. healthy controls. Their results also showed that iRBD patients with negative asyn SAA results were less likely to develop a synucleinopathy compared to patients with detected asyn seeds at 2, 4, 6, 8, or 10 year follow-up (Iranzo et al., 2021). Detection of asyn seeds in iRBD patients have also been investigated by other researches showing a similar ability of asyn SAA to detect asyn seeds prior to clinical diagnosis of PD (Poggiolini et al., 2022).

### Disease Stratification

The ability of asyn SAAs to discriminate between synucleinopathies has also been investigated, though with mixed results. While the asyn SAA is mostly used as a binary readout of disease status, attempts have been made to differentiate patients by the kinetic parameters of asyn aggregation. In a cohort of PD and DLB patients with different genetic mutations, researchers were able to show association between seeding profile and genetic status (Brockmann et al., 2021). Importantly, the authors also looked at repeated longitudinal measurements of 86 PD patients, noting that kinetic parameters remain stable over a timeframe of 7 years. This suggests that seeding activity is an attribute of the disease and not a marker that changes with disease duration. In a different study, Poggiolini et al. (2022) showed correlation between kinetic parameters and disease severity in a group of 24 MSA patients, though found no correlation in PD patients. While some groups have shown little to no seeding capacity of CSF samples from MSA with 6 or 32% sensitivity (Shahnawaz et al., 2020; Poggiolini et al., 2022), others have shown considerably higher seeding capacity with 75 or 96% sensitivity (van Rumund et al., 2019; Rossi et al., 2020). A possible explanation could be that differing experimental conditions between the studies account for this variability. Indeed, recent evidence suggests that the reaction buffer composition impacts the ability to detect MSA seeds in brain homogenates (Martinez-Valbuena et al., 2022). The maximal ThT fluorescence signal is characterized by high sensitivity in both studies, but with a much lower signal in MSA compared to PD. This difference is presumably due to conformational differences in the MSA and PD derived aggregates leading to a differential ThT binding.

### Limitations and Challenges

Over the years several labs have independently established and named their asyn SAA methods. Consequently, despite using an overall similar set-up, there is a wide range of different asyn SAA protocols with slight variations in monomeric protein, reaction conditions, shaking conditions, temperature etc. These parameters can be critical for the outcome of the assay. For instance, the time needed for the detection of seeded asyn by pathological CSF samples using SAA varies between 15 and 220 h (Fairfoul et al., 2016; Shahnawaz et al., 2017; Groveman et al., 2018). Apart from the time-course differences, there is also variability of the fluorescence amplitude between SAA reactions for the same samples (Russo et al., 2021). Therefore, differences in procedures, reaction conditions and reagents used in SAA protocols can all affect the process of asyn aggregation.

In SAA, the samples are tested in triplicates or quadruplicates to increase the assay's specificity. However, there is often variability between the technical replicates of the same sample, complicating analysis. It can be partially attributed to the non-linearity of the asyn aggregation process, which also depends on the concentration and conformation of the seeds, the asyn monomer preparation and the presence of other molecules in the biological samples that may affect the reaction kinetics. In addition, the breaking of asyn oligomers and the re-seeding of asyn is a very unpredictable process that could be highly variable, especially when it takes place in a reaction that can be influenced by numerous factors. Indeed, there is currently no consensus about exactly how many technical replicates to perform, and how many needs to be positive for an inconclusive, negative or positive readout. Notably, this assay variability might confer biological information as the number of positive technical replicates out of four have been shown to correlate with genetic status and motor and cognitive impairment in PD patients (Brockmann et al., 2021).

The microenvironment of the reaction is important for the aggregation process and therefore its dependence on the sample matrix is high. The composition of CSF samples, which are mostly used for SAAs, may vary between individuals in terms of protein concentration and matrix constitution in general. CSF has indeed been found to inhibit the asyn amplification reaction (Shahnawaz et al., 2017). One such example of inhibitory matrix could be from red blood cells in CSF samples, due to contamination during lumbar puncture, as this was found to have an inhibitory effect on PrP^Sc^ seeding activity, resulting in false negative responses from the RT-QuIC assay (Cramm et al., 2016; Foutz et al., 2017). In contrast to red blood cells, high total protein concentrations and raised white cell counts in CSF samples have been found to lead to false positive SAA results (Green, 2019). In more complex samples such as brain tissue, more dilution is needed compared to CSF to dilute the inhibitory matrix affecting the amyloid formation (Hoover et al., 2017). Where CSF is often used undiluted in asyn SAA, tissue homogenates are diluted ~10^3^–10^5^ (Fairfoul et al., 2016; Groveman et al., 2018; Shahnawaz et al., 2020; Bargar et al., 2021).

Formalin fixed and parafin embedded (FFPE) samples also show potential as material for asyn SAAs, as studies in skin (Manne et al., 2020) and submandibular glands (Manne et al., 2020) have shown ability to differentiate PD patients from control. However, both tissue types show lower sensitivity (75–75%) compared to frozen samples (96–100%), possibly due to a suppressing effect of formalin fixation on the seeding capacity.

Most animal studies using asyn SAA methods are confined to prion disease research (reviewed by Collins and Sarros, 2016 and Atarashi, 2022). Recent successful use of animal tissue for the conversion of human asyn substrate also strengthens the position of the asyn SAA as a robust research tool. In a study of transgenic mice expressing human αsyn with A53T mutation, brain homogenates were able to seed A53T recombinant asyn substrate in a SAA assay, but not WT recombinant asyn (Han et al., 2020). The same mouse model was also used to shown presence of seeding activity of colon tissue in mice as early as in 3-month-old mice, months before detectable seeding activity in brain (Han et al., 2021).

Despite its challenges, the asyn SAA seems to be the best method up to date to explore pathology in biopsies and fluids for early diagnosis and patient stratification. To increase diagnostic value, the asyn SAA could also be used in combination with more conformation-specific dyes than ThT.

## Luminescent Conjugated Oligothiophenes to Identify Different Strains

### LCO Assay

Although amyloid fibrils have been studied using established protein-characterization techniques throughout the years, their oligomeric precursor states require sensitive detection in real-time. A new class of ultra-sensitive dyes, denoted LCOs, initially developed for characterization of prion protein and amyloid, is recently also available to study the molecular architecture of asyn protein. LCOs bind to the repetitive cross–β-sheet structures of pathogenic protein aggregates and display spectral differences based on the twisting of the flexible LCO backbone. Thus, the distinct conformation or “spectral fingerprint” of the ligand upon interaction with a certain asyn aggregate reflect the 3D structure of that asyn aggregate (Klingstedt and Nilsson, 2012; Gustafsson et al., 2021). These fluorescent LCO-ligands thus identify a broader subset of pathogenic protein aggregates than conventional ligands such as ThT, as they have been established as a class of ligands for superior recognition and spectral assignment of disease-associated protein aggregates, including different polymorphic Aβ (Ellingsen et al., 2013; Rasmussen et al., 2017; Calvo-Rodriguez et al., 2019; Lantz et al., 2020; Liu et al., 2021), asyn (Klingstedt et al., 2019; Shahnawaz et al., 2020) aggregates, as well as toxic and non-toxic polymorphic variants of insulin, both *in vitro* (Psonka-Antonczyk et al., 2012; Mori et al., 2021) and in patient biopsies (Yuzu et al., 2020).

Owing to their electronically delocalized conjugated thiophene backbones, LCOs exhibit intrinsic conformational dependent fluorescence characteristics that can be recorded by different modes of detection such as hyperspectral- and fluorescence life-time microscopy (Lantz et al., 2020; Gustafsson et al., 2021). Since development of the first ligands, the founding lab has generated a library of chemically diverse thiophene-based ligands that can bind to distinct aggregate conformers. Combining a variety of ligands enables the detection and morphological characterization of many different types of asyn-aggregate morphotypes with high accuracy. A study using a set of 7 ligands has demonstrated successful stratification between PD and MSA (Shahnawaz et al., 2020). Different ligands can be created by replacing selected thiophene motifs with other heterocyclic building blocks, and by introducing a variety of side-chain functionalities along the conjugated thiophene backbone. This design strategy has been successful for achieving aggregate specific diversity of the ligands (Bäck et al., 2016; Shirani et al., 2017; Klingstedt et al., 2021).

### Disease Stratification

Similar to asyn SAA, LCOs have mainly been used in prion disease; and also in an AD context. As with synucleinopathies, AD patients exhibit a heterogeneous clinical profile, despite a rather homogeneous Aβ distribution in the neocortex of the brain across patients. Thus, also in AD the variable phenotype amongst patients may be associated with the presence of different Aβ strains. Liu et al. (2021) observed a high conformational diversity of Aβ in the neocortex of AD cases, with most distinct spectral profiles in the temporal cortex of cases with shorter disease duration, implicating distinct Aβ strains are responsible for more rapidly progressing AD. Moreover, Rasmussen et al. (2017) observed distinct spectral fingerprints of different amyloid aggregates within a single end-stage AD brain. These studies indicate the presence of an amyloid “strain cloud” with a variable mix of aggregates and their emission spectra, rather than a single strain causing disease phenotype. The authors speculate that amyloid distribution may be more complex at late disease stages, and that LCO spectra may yield better stratification value at earlier disease stages (Rasmussen et al., 2017).

More recently, conformation-specific LCO ligands have also been used to differentiate between synucleinopathies. Since it has been reported that oligodendrocytes, but not neurons, can convert asyn into a conformer related to GCIs (Peng et al., 2018), most studies focused on differentiating PD and MSA. Klingstedt et al. observed a shift in the HS-68 ligand emission spectrum at wavelengths of 486 and 573 nm for PD- and MSA-derived asyn, respectively, hereby successfully distinguishing PD and MSA cases. Additionally, they showed a differential fluorescence decay of the h-FTAA ligand when binding to PD- vs. MSA-derived aggregates, nicely illustrating that LCOs can interact in different ways with asyn aggregates in PD and MSA (Klingstedt et al., 2019). Furthermore, stratification potential can be optimized by combining the LCO and asyn SAA assays. Shahwanaz et al. applied a panel of seven LCO ligands on asyn-SAA-amplified CSF- and brain-extracted asyn from PD and MSA patients. They observed differential binding capacity of the ligands to PD- vs. MSA-derived asyn. Specifically, the ligand HS-199 appeared to bind preferentially to PD- but not MSA-derived asyn, and opposite, the HS-169 ligand seemed to favor MSA over PD aggregates. Results were similar between brain- and CSF-derived asyn, indicating strain morphology is similar in brain and CSF (Shahnawaz et al., 2020). Taken together, both studies support the use of LCO-ligands to distinguish between synucleinopathies. The combination of both LCO and asyn SAA may further increase diagnostic value.

The affinity and conformational sensitivity of these ligands has also been investigated in rodent models of neurodegenerative disease. Multiple studies have shown that LCOs are able to differentiate structurally distinct prion strains in rodent models of prion disease (Sigurdson et al., 2007; Magnusson et al., 2014; Aguilar-Calvo et al., 2020) and AD (Nyström et al., 2013; Klingstedt et al., 2015). Interestingly, intensity shifts were found in the HS-68 spectra of Aβ and tau aggregates in the same brain area of young and old transgenic AD mice (Nyström et al., 2013; Klingstedt et al., 2015). The superior functionality of this ligand is attributed to distinct spacing between the anionic groups along the conjugated backbone (Klingstedt et al., 2015). The cellular environment is subject to age-related changes, therefore it is not surprising that age-dependent spectral differences have been observed in these models. Similarly, we observed age-dependent differences in aggregate morphology (size and density) in PD wild-type rodents visualized with regular immunohistochemistry (IHC) against asyn pathology. Moreover, pathology was more resistant to proteinase K pretreatment in old rodents, indicating more rapid maturation of pathology in old subjects (Van Den Berge et al., 2021). Importantly, we previously reported morphological differences in aggregated asyn in body-first and brain-first asyn-seeded rodents, and across organs, with regular IHC (Van Den Berge and Ulusoy, 2022). Although we cannot reliably differentiate between body-first and brain-first-type pathology using only IHC, these results do indicate that pathogenic asyn may possess different conformations depending on the cellular environment it is formed in, which is also influenced by age. How these morphological differences are linked to the molecular architecture of asyn remains to be investigated.

### Future Applications

Now that a growing number of asyn-specific ligands are being developed, future animal and patient research should investigate whether the morphological changes observed with regular IHC are associated with changes in the molecular architecture of the different asyn strains in the “strain cloud” of different synucleinopathies. Moreover, the effect of age on strain architecture should be investigated. Such information may be useful to predict the disease progression more accurately depending on the age at diagnosis.

In animal studies, pathogenic asyn fibrils can be injected in the gut or brain to investigate bidirectional propagation of pathology through the autonomic connectome (Van Den Berge and Ulusoy, 2022). Tissue samples from various sites of the PNS and CNS could then be collected to assess the presence of aggregated asyn (using regular immunostainings) as well as to distinguish between the aggregate morphotypes (using conformation-specific LCO-ligands). These ligands could help elucidate whether the conformation of the aggregated asyn changes throughout the autonomic connectome during pathology propagation. If morphological changes are observed it would be in support of the hypothesis that micro-environmental variations in the various cell types along the gut-brain axis may affect the conformation of asyn strains.

**Figure 4:**
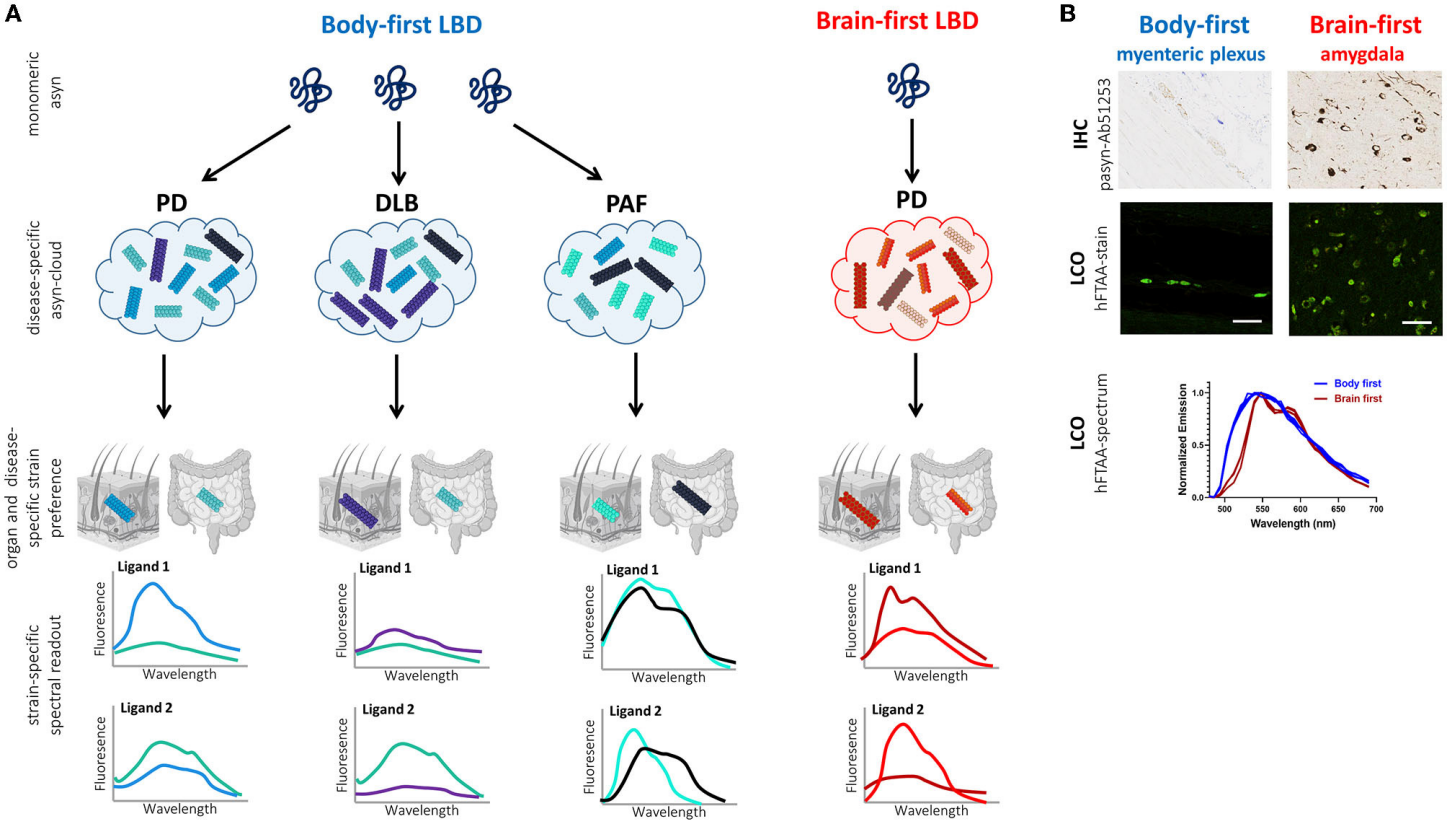
Working principle of LCO-mediated asyn-strain characterization in LBDs. **(A)** The strain hypothesis applied to body-first and brain-first LBDs: monomeric asyn may misfold into any pathogenic conformer (influenced by the cellular milieu at the disease onset site), which is responsible for determining which LBD-subtype a patient develops: typical body-first PD (with pre-motor iRBD), DLB, PAF, or brain-first PD (without pre-motor RBD). Due to bidirectional trans-synaptic spread, we hypothesize that each LBD is characterized by a subtype-specific mix of oligomeric and protofibrillar species, the subtype-specific “asyn cloud”, responsible for its phenotype. Furthermore, the cellular environment in a certain organ may favor one conformer over the other from a patients' asyn cloud. Consequently, the spectral fingerprint of pathology in peripheral biopsies likely differs across organs (such as skin and gut) and LBD-subtypes. A combination of multiple fluorescent LCO-ligands on gut and skin biopsies may enable more accurate stratification in pre-motor disease stages, limiting late and misdiagnosis, paving the way for more accurate prognosis and personalized treatment. Furthermore, we speculate that DLB-subtypes may have a more similar cloud distribution, compared to MSA, since asyn in MSA is mainly confined to oligodendrocytes and not neurons/axons. **(B)** Histology and LCO data in the gut (myenteric plexus) and amygdala of a gut- and brain-first wild-type rodent model, respectively. Immunohistochemistry (IHC) against phosphorylated asyn (Ab51253) is unable to differentiate between gut and brain pathology. In contrast, the LCO assay using the hFTAA-ligand shows a clear difference in the spectrum of gut- and brain-derived aggregates with a more typical two-peak hFTAA spectrum in the brain, indicating gut and brain pathology possess different structural characteristics despite using the same seeds for disease initiation (preliminary data). Scalebar: 50 μ*m*.

Figure 4 illustrates the working principle of LCO-mediated strain characterization in gut and skin biopsies of body- and brain-first LBD patients, possibly enabling early patient stratification. Preliminary data from our group shows organ-specific differences in the spectrum of hFTAA-ligand binding to pathogenic asyn in enteric vs. amygdala neurons. Since PD pathology was initiated using the same asyn strain, these findings indicate that the cellular environment at the disease initiation site may impact asyn strain morphology, which may be responsible for phenotypic differences between body-first and brain-first PD already at very early disease stages, supporting that LCO stratification potential is probably higher at immature disease stages when the “strain cloud” is still confined to one dominant strain (per organ).

Finally, LCOs may be used to assess the seeding activity and morphology of different asyn strains in cell models. The distinct fluorescence characteristics and the specific binding modes of the different LCOs can be employed to isolate a specific asyn strain from a tissue sample. Tissue lysates can be fractionated with conventional techniques, such as sucrose gradient fractionation and non-denaturing gradient gel electrophoresis (Jackson et al., 2016; Morgan et al., 2020). Following, the ligand of interest can be coupled to magnetic beads or resins to selectively capture asyn aggregates from homogenates/lysates (from for example a patient's gut or skin biopsy) based on the ligand's selective binding mode. The isolated asyn strain can then be used to investigate its seeding amplification and other structural properties in cell models (Falcon et al., 2015; Morgan et al., 2020). Cells expressing human asyn will form new aggregates upon exposure to the isolated asyn strain. These newly formed aggregates may be screened with a broad range of ligands in combination with live-cell imaging to discern the time course and spatial distribution of seeding and propagation of a distinct strain. Furthermore, the ligands can be subsequently used for super-resolution fluorescence imaging (Ries et al., 2013) in order to achieve high-resolution information concerning the location of distinct asyn aggregates in relation to specific cellular organelles.

## Conclusion and Future Perspectives

Based on the existing literature we hypothesize that the cellular environment of the predominant neuronal subtype at the primary inoculation site (for example, enteric neurons in body-first LBD vs. amygdala neurons in brain-first LBD) may determine the dominant strain in the subtype-specific asyn cloud. Trans-synaptic bidirectional spread through the autonomic connectome may further add different conformers to a patient's asyn cloud. Furthermore, we speculate that LBD-subtypes may have a more similar cloud distribution than MSA as pathology is confined to different cell-types (neurons vs. oligodendrocytes).

The long prodromal phase of LBD provides an opportunity for early intervention, though this necessitates methods for early diagnosis. Imaging autonomic dysfunction enables pre-motor diagnosis of body-first LBD, however, it requires extensive damage to the ANS. Ideally, we would want to diagnose in the prodromal phase, before pathology built-up was able to cause extensive damage. Classic histological methods are not able to detect immature pathology, nor are able to differentiate between disease-specific strains. More recent methods such as asyn SAA and conformation-specific LCO-ligands are able to characterize seeding and structural properties of disease-specific strains. Despite its challenges, the asyn SAA seems to be the best method to date to explore pathology in biopsies and fluids for early diagnosis and patient stratification in synucleinopathies. To increase its diagnostic value, asyn SAA can be used in combination with the more sensitive LCO-ligands. The fact that PD- and MSA-derived asyn aggregates differ in LCO-ligand stainability, emission profiles and fluorescence lifetimes, nicely illustrates the stratification potential of LCO.

The LCO assay has mainly been used in the context of prion disease and AD. Future studies should focus on the characterization of different dominant asyn strains across synucleinopathies in patients and modeled in animals. Application of a LCO-ligand panel to biopsies and fluid samples could classify a certain dominant asyn strain to be associated with a certain synucleinopathy, revealing different potential therapeutic targets and facilitating the development of disease-specific treatments, such as antibodies for passive immunization. Antibodies could be tailored to combat uptake and propagation of a certain disease-specific asyn strain. If multiple dominant strains would be detected in a patient, treatment may require a personalized cocktail of several antibodies (Folke et al., 2022). Thus, the ability of the LCO-assay to differentiate morphotypes of aggregated asyn in easily accessible tissue and fluids would allow personalized treatment at early phases of disease, potentially prior to autonomic and CNS damage, altogether highlighting the importance of this assay in early disease-specific diagnosis.

Disease-modifying treatment strategies such as immunotherapy have been suboptimal in clinical trials compared to preclinical conditions, as described by Folke et al. (2022). The authors discuss that screening of antibody-treatment in brain-first only LBD models as well as suboptimal patient selection in clinical trials, may have contributed to disappointing translational results. We speculate that a monoclonal antibody-treatment, optimized in an animal model exhibiting a single asyn strain, may not be effective in a patient characterized by multiple asyn strains, which limits translational outcome. The discovery of several distinct asyn conformers, using a library of conformation-specific LCO-ligands in peripheral biopsies of a certain LBD-subtype, may reveal a patient's asyn cloud, paving the way for personalized immunotherapy with a mix of conformation-specific antibodies in the treatment. Thus, there is a need for continuous synthesis of new ligands specific for asyn conformers. Such ligands will be vital for evaluating novel therapeutic strategies for synucleinopathies and enhance translation ability.

Finally, the several hypotheses raised in this review remain to be proven. Future body-first and brain-first LBD animal models should investigate whether asyn strain characteristics change during trans-synaptic spread through the autonomic connectome, and whether the cellular environment where the very first asyn seed is created, dominates the subtype's cloud distribution. Such studies should further aim to demonstrate whether an accurate assessment can be made of the strain mix present in peripheral biopsies, prior to autonomic and CNS deficit.

## Data Availability Statement

The original contributions presented in the study are included in the article/supplementary material, further inquiries can be directed to the corresponding author.

## Author Contributions

MJ and NV contributed equally to the abstract, text, and figures in paragraphs 1, 2, 3, 5, and 6. HG, VT, and P-HJ contributed equally to paragraph 4. KN and ML contributed equally to paragraph 5. KK and PB contributed equally to paragraph 3. All authors contributed to the article and approved the submitted version.

## Funding

NV is funded by the Lundbeck Foundation (R322-2019-2544), Danish Parkinson's Association, Bjarne Saxhofs Foundation and Jascha Foundation. MJ and PB are funded by the Lundbeck Foundation(R276-2018-294).

## Conflict of Interest

The authors declare that the research was conducted in the absence of any commercial or financial relationships that could be construed as a potential conflict of interest.

## Publisher's Note

All claims expressed in this article are solely those of the authors and do not necessarily represent those of their affiliated organizations, or those of the publisher, the editors and the reviewers. Any product that may be evaluated in this article, or claim that may be made by its manufacturer, is not guaranteed or endorsed by the publisher.
